# The effects of mouse ovarian granulosa cell function and related gene expression by suppressing BMP/Smad signaling pathway

**DOI:** 10.1080/19768354.2018.1497706

**Published:** 2018-09-19

**Authors:** Li Zhang, Hejian Wang, Daolun Yu, Jie Chen, Chaofeng Xing, Jie Li, Jun Li, Yafei Cai

**Affiliations:** a College of Life Science, Anhui provincial Key Lab of the Conservation and Exploitation of Biological Resources, Anhui Normal University, Wuhu, People's Republic of China; b College of Animal Science and Technology, Nanjing Agricultural University, Nanjing, People's Republic of China

**Keywords:** Granulosa cells, BMP/Smad signal pathway, Smad9, BMP-4

## Abstract

BMP I type receptor inhibitor can selectively inhibit BMP/Smad signaling pathways, mainly by inhibiting the BMP I type receptor activity to prevent phosphorylation of Smad1, Smad5 and Smad9. The aim of the present study was to explore the effects of mouse ovarian granulosa cell function and related gene expression by suppressing BMP/Smad signaling pathway with LDN-193189(A type of BMP I type receptor inhibitor). In this study, we cultivate the original generation of mouse ovarian granular cells then collect cells and cell culture medium after treatment. Cellular localization and expression of Smad9 and P-smad9 proteins was studied by immunofluorescence (IF) in the ovarian granulosa cells of mouse; Related genes mRNA and proteins expression was checked by QRT-PCR and Western blot; Detected the concentration of related hormones by using ELISA kit; finally, the growth of the cells was analyzed by plotting cell growth curve with CCK-8 assay. The results indicate that, suppression of BMP/Smad signaling pathway can inhibit the expression of LHR and FSHR, inhibit cell proliferation and decrease E_2_ secretion, the mechanism of action maybe reduce the expression of smad9, at the same time, we found that the feedback regulation of smad9 may affect the expression of FSHR and cell proliferation.

## Introduction

The steroid hormones secreted by ovarian granule cells (GCs) include progesterone, estradiol, luteinizing hormone, follicle-stimulating hormone, and aromatase cytochrome P450. Among them, the primary physiological function in regulation is E_2_ and P_4_, they can regulate the structure and function of the female reproductive system, affect the apoptosis of the ovarian GCs, maintain the normal development of follicles in the ovary and regulate the development potential of the embryo. As the main functional cells in the ovaries, GCs play a crucial role in the maturation of oocytes, it affects the proliferation and differentiation of the ovarian directly.

Bone morphogenetic proteins (BMPs) belong to the transforming growth factor β (TGF-β) superfamily. Recently, the role of BMPs in reproduction has been researched extensively, including to follicular growth and development, GCs proliferation and oocyte, ovulation and luteinization. Among the more than 20 known members of the BMP family, eight members were recognized to play an important role in the maturation of oocytes and the growth and development of ovaries (Table 1) (Onichtchouk et al. 1999; Henningfeld et al. 2002; Yokota & Mori 2002; Shimasaki et al. 2004; Drummond 2005), including BMP-2, BMP-3, BMP-4, BMP-5, BMP-6, BMP-7, BMP-15 and GDF-9(Growth differentiation factor-9) (Shimasaki et al. 1999; Shimizu et al. 2004; Brankin et al. 2005; McNatty et al. 2005; Pierre et al. 2005). The expression of BMP-4 in follicular GCs can support follicular selection and promote differentiation (Kim et al. 2013). Studies have shown that BMP-4 proteins are expressed in ovarian GCs, theca cells and oocytes, and the expression level in all levels of follicle is different, change along with the development of follicles (Nilsson & Skinner 2003; Drummond 2005). It plays an important role in the maturation of oocyte and the growth and development of ovaries (Shimasaki et al. 2004; Shimizu et al. 2004; Juengel & McNatty 2005; Gilchrist et al. 2008).

**Table 1.** Regulation of BMPs to steroidogenesis and cell proliferation in folliculogenesis.

	P450scc	E_2_	Number of cells
BMP-2	?	↓	↑
BMP-4	↓	–	?
BMP-5	?	–	↑
BMP-6	↓	↓	↑
BMP-7	–	?	↑?
BMP-15	↓	?	↑
GDF-9	↑?	↑	↑

Note: ↑, stimulate; ↓, inhibit; – no change; ?, unknow; ↑?, possibly stimulate.

**Figure 1.**
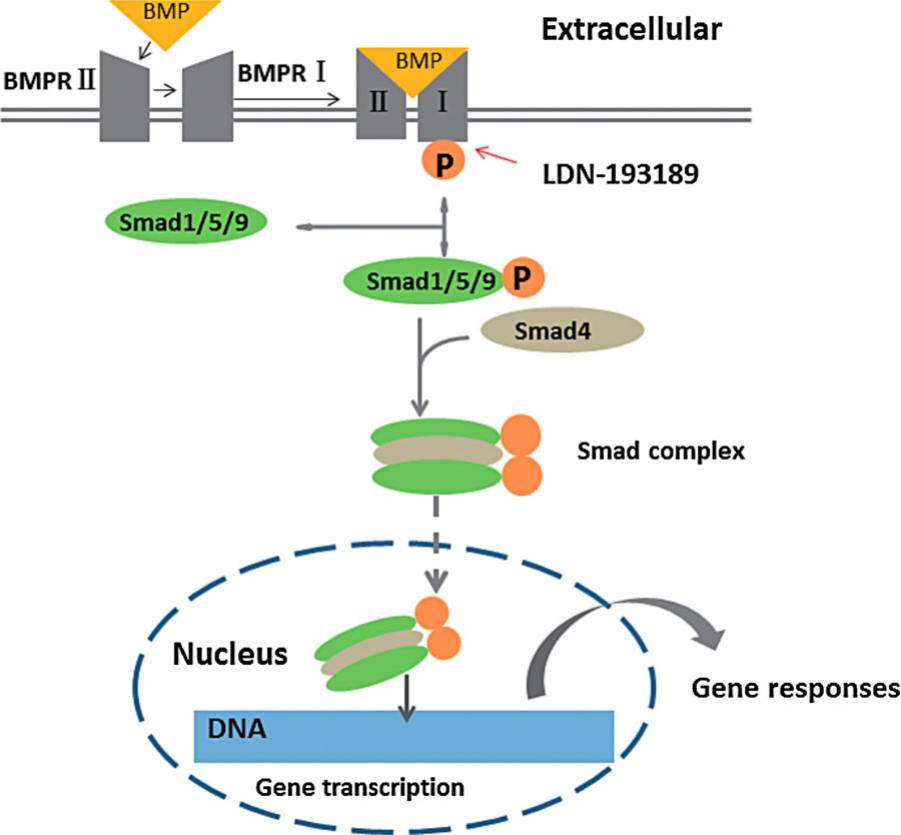
BMP/Smad signaling pathway. Note: P, phosphorylation; BMPR, BMP receptor.

BMP/Smad signaling pathway plays an important role in the occurrence of follicles (Glister et al. 2004). Smads are the major signal transducers for the TGF-β family receptors (Derynck & Zhang 2003). Smads proteins transmit the BMPs extracellular signal from the cell membrane into the nucleus via regulating factor with different transcription proteins or its transcriptional regulatory factor, and different Smads mediate the signal transduction of different TGF-β family members (Pangas 2012; Runyan et al. 2012; Wan et al. 2012). SMAD proteins have found eight members in mammals: smad1-9(also known as Smad8), they were classified into three subgroups based on their correspondence and requests: R-smads, Co-smad and I-smads. Among them, Smad1/5/9 participated in BMPs signal transduction (Chen et al. 2007). Lots of experiment has proved that the channel model is: the TGF-β ligand first own II type receptors on cell membrane (Figure 1), then phosphorylation activation type I receptor, forming complexes, make it in target cells and membrane receptor coupling of GSs activation, the activation of TGF-β with Smads molecules combine to form complex signal, the complex and immediately transferred to the nucleus in interaction with various transcription factors, regulate the transcription of related genes, and mediated TGF-β on a cellular level of biological effects of regulating cell growth. Recent studies have found that Smad9 is a new type of transcriptional regulator in bone morphogenetic protein signaling (Tsukamoto et al. 2014). However, the study on the function of Smad9 protein in ovarian GCs is rare, and the specific mechanism and signal pathway are unknown.

This study based on cultured mouse ovarian GCs in vitro, the effect of Smad9 on gene expression related to ovulation and its effect on the proliferation of mouse ovary GCs were studied for the first time.

## Materials and methods

### Experimental animal

Female SPF-KM mice (Eight weeks old and weight around 30–35 g) were purchased from the Qinglongshan Laboratory Animal Center (Nanjing, China). Mice were raised in clean animal cages, keeping free diet. The room is kept in a constant temperature (25°C) and 12–12 h light–dark cycles. All animal experiments were complied with the ARRIVE guidelines and carried out in the National Institutes of Health guide for the care and use of laboratory animals. All animal experiments were approved by the Anhui Normal University Academic Ethics Committee.

### Preparation of primary GCs

Mice were injected with PMSG(10IU, Ningbo second hormone factory) to increase GCs number, and HCG (10IU, Ningbo Sansheng Pharmaceutical Co., Ltd.) was injected after 48 hours later (Elvin et al. 1999; Park et al. 2010), then kill the mouse after 24 hours. Ovaries were removed and the GCs were extracted by puncturing ovaries. Collected GCs were centrifuged by Trypsin Solution (Sangon Biotech) (37 ^o^C, 3 min), then add DMED Medium(Sangon Biotech) with 10% FBS and 1% Penicillin–streptomycin solution (Sangon Biotech) into the cell suspension. GCs were maintained at 37°C in a humidified atmosphere containing 5% CO_2_. Cells that have the same growth were randomly divided into four groups: control group, BMP-4 group, LDN-193189 group and BMP-4 + LDN-193189 group.

### Immunofluorescence staining

Localization and expression of Smad9 and P-smad9 proteins in the GCs was evaluated by immunofluorescence (IF). Cells growing on the glass slide were washed in PBS, fixed in Immunol Staining Fix Solution (Beyotime Biotechnology) for 10 min, permeabilize with 0.5% Triton for 10 min, after that, blocked for 1 h at room temperature with 5% BSA. Then the GCs were incubated with antibodies against Smad9 (1:1000), P-smad9 (1:800) at 4°C for overnight. Cells were rinsed three times in PBS after primary antibody incubation and then were sequentially incubated with Alexa Fluor 555 donkey anti-rabbit secondary antibody (Beyotime Biotechnology, 1:1000) (Nie et al. 2012) for 1 h at room temperature. Finally, cells washed in PBS to omitting the antibodies mixture. After that, we used anti-fade Mounting Medium with DAPI (Solarbio, Beijing, China) to mount slides. Fluorescence images were acquired with an Olympus BX61 fluorescence microscope. Fluorescence images were recorded using a 40× objective lens.

### Quantitative real-time PCR (qRT-PCR)

Total RNA was extracted by using Trizol reagent (Invitrogen, Carlsbad, CA, USA), isolated RNA were reversely transcribed based on the Fast Quant RT Kit (Tiangen, Beijing, China). Amplification and real-time detection were performed on IQ5 instrument (Bio-Rad) by using the Super Real PreMix Plus (Tiangen). The improved four steps reaction were used as follows: 95°C 15 min; 95°C 10 s, 60°C 32 s, 72°C 32 s, 85°C 6 s, for 40 cycles; the melting curve analysis ranging from 60°C to 95°C, gradually increasing at a speedy of 0.5°C every 10 s. Relative quantitative analysis of the final results was normalized to GAPDH by using the 2^−ΔΔCt^ method. The primers used for mice were as Table 2.

**Table 2.** Details of PCR primers employed.

Genes	Primer sequence (5′ to3′)
GAPDH	AGGTCGGTGTGAACGGATTTG TGTAGACCATGTAGTTGAGGTCA
Smad1	CAGCTACTGGCGCAGTCTGT
	ACATCCTGCCGGTGGTATTC
Smad5	TGCTCAGCTTCTGGCTCAGT
	GTGACGTCCTGTCGGTGGTA
Smad9	CGATCATTCCATGAAGCTGACAA TGGGCAAGCCAAACCGATA
CYP19a1	GCCTGTTGTGGACTTGGTCA
	ACTCGAGCCTGTGCATTCTTC
PRLR	ATCCACAAATGTCGTTCCCCT TGGAAGTGTACTGCTTGCTAAAG
FSHR	TGCTCTAACAGGGTCTTCCTC TCTCAGTTCAATGGCGTTCCG
LHR	AATGAGTCCATCACGCTGAAAC CCTGCAATTTGGTGGAAGAGA

### Western blot

Cells were lysed in RIPA buffer to extract the protein samples on the ice, and then measured protein concentration with bicinchoninic acid (BCA) protein assay kit (Thermo Scientific), denatured protein prepare loading quantity of sample, equal amounts of proteins were fractionated onto SDS-PAGE gels, transmembrane, then blocked with 5% non-fat milk in TBST and probed with primary antibody 4°C overnight and the corresponding secondary antibody for 1 h at the room temperature. Finally, films were developed and fixed in the dark room. GAPDH, Smad9, and P-smad9 antibodies were procured from ZSGB-Bio.

### Hormone analysis

After cell drug treatment, cell culture supernatants were taken to detect hormone levels based on Enzyme-Linked Immunosorbent Assay (ELISA) kit (Halin, China).

### Cell proliferation assay

Cell growth was determined using the cell counting kit-8 (CCK-8) reagent (Beyotime Biotechnology). Adjust the cell concentration to 1 × 10^4^, add 100 ul of mixed cell suspension to 96-well plates. As instructed in the manual, stimulated with the indicated chemicals and then incubated with the CCK-8 reagent. Absorbance was measured at 490/630 nm on a microplate reader, the time interval is six hours.

### Statistical analysis

The experiment data apply SPSS version 20.0 (IBM, Armonk, NY, USA) to statistical analysis. We set up the significant level as *α* = 0.05, when *P < .05, it was considered statistically significant. Measurement data was showed mean ± SEM. The compared data apply to the one-way analysis of variance. Compared between groups was used the LSD method, when comparing differences between the groups was statistically significant.

## Results

### LDN-193189 affects the expression and localization of smad9 and P-smad9 proteins

**Figure 2.**
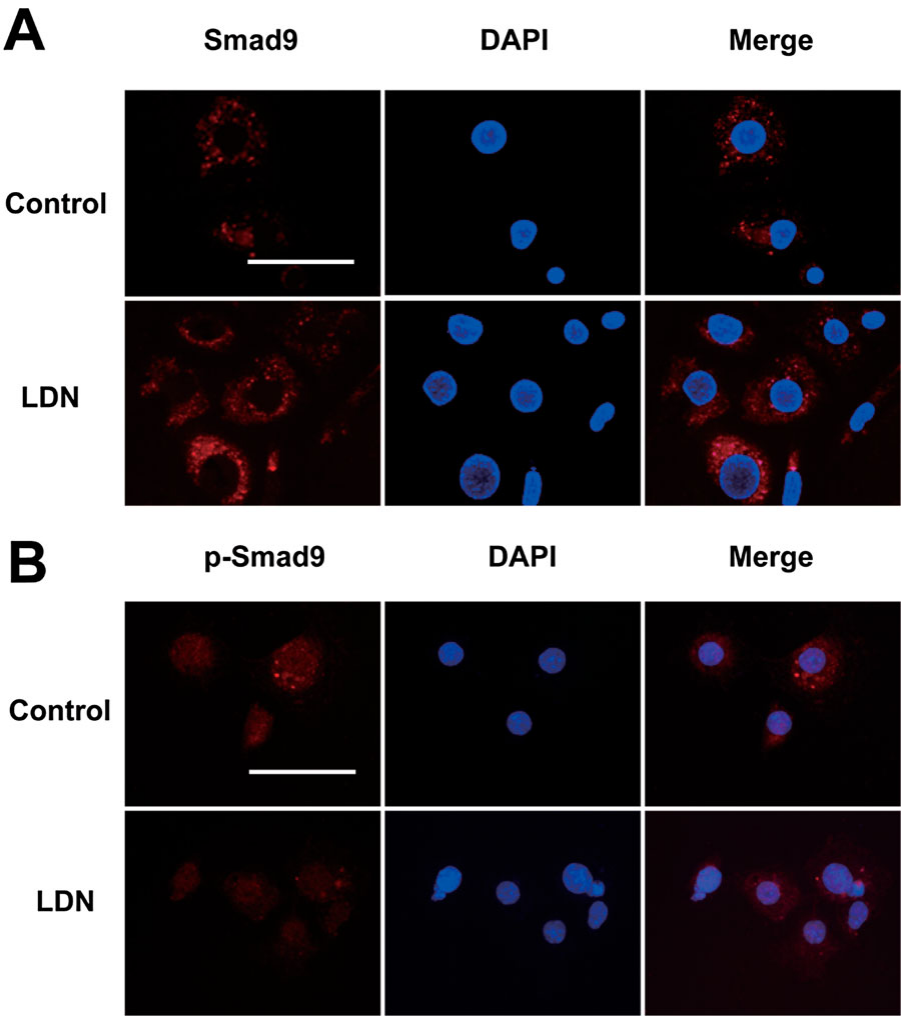
Immunofluorescence analysis of Smad9 (A) and P-smad9 (B) location (red) after LDN-193189(100 nM) treatment. DAPI stain is shown in blue. (Original magnification ×400), Scale bar = 100 μm. Data are presented as means ± S.D. of three independent experiments.

To determine the effect of LDN-193189 on cellular localization of smad9 and P-smad9, IF was used on the GCs. Smad9 proteins only exist in the cytoplasm of ovary GCs, P-smad9 proteins are expressed in the cytoplasm and nucleus of ovary GCs. In this study, we divided the cells into two groups, one is control group, another group of cells was treated with LDN-193189 (a kinase inhibitor of BMP type I receptors). The detection indicated that, after treating by 100 nM LDN-193189, the expression of Smad9 proteins in cytoplasm are increased (Figure 2(A)), compare with control group, the expression of P-smad9 proteins are decreased (Figure 2(B)). IF results show that under the action of exogenous LDN-193189, the phosphorylation levels of smad9 protein in the ovarian GCs were significantly decreased, it is suggested that Smad9 may participate in the regulation of the apoptosis of ovarian granulosa through the classical BMP/Smad signaling pathway.

### LDN-193189 increases the expression of smad9 by inhibiting BMP/Smad signaling pathway

**Figure 3.**
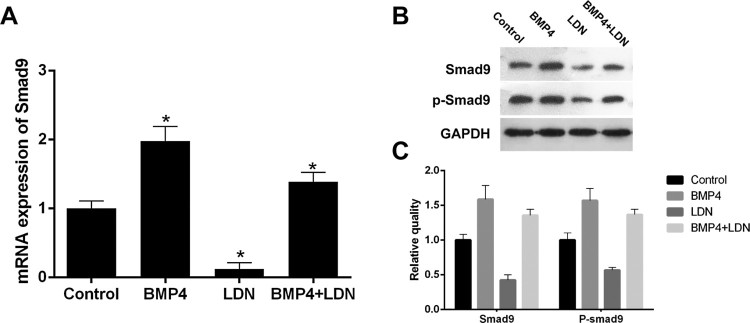
Expression of Smad9 and P-smad9 proteins and mRNAs in mouse ovary granule cells. (A) Western blot analysis of the whole cell extracts that were prepared from ovary granule cells that were stimulated with or without BMP-4 100 (μg/μl) and LDN-193189 (100 nM) for 48 h. (B) Quantitative analysis of data in A. Protein levels of Smad9 and p-smad9 protein were assessed by Western blot. (C) Relative Smad9 mRNA levels. Total RNA was extracted from mouse ovary granule cells and subjected to QRT-PCR to determine the mRNA levels. Data were normalized to Gapdh mRNA levels. Data are expressed as the mean ± S.D. (*N* = 3), *P < .05.

To explore the effect on gene expression of smad9 by inhibit signaling pathway, we divided the cells into four groups (control group, BMP-4 group, LDN-193189 group and Bmp-4 + LDN-193189 group), cells’ RNA and protein were collected for Qpcr (Figure 3(A)) and western blot (Figure 3(B,C)). Experimental results show that, BMP-4 can promote the expression of Smad9 significantly, (*P* < .05), compare with control group, Smad9 mRNA expression was significantly reduced after treat by 100 nM LDN-193189 (*P* < .05), also, Bmp-4 + LDN-193189 group had higher protein expression than control group, but it is lower than BMP-4 group (*P* < .05), and there was no significant difference with the control group (A). Western Blot shows that BMP-4 promotes the expression of Smad9 protein, but LDN-193189 reduces the expression of proteins. The protein level in Bmp-4 + LDN-193189 group is lower than control group and BMP-4 group, but it is higher than LDN-193189 group. The expression of P-smad9 protein was similar to smad9 (B and C).

### The secretion of hormones affects by inhibiting BMP/Smad signaling pathway

**Figure 4.**
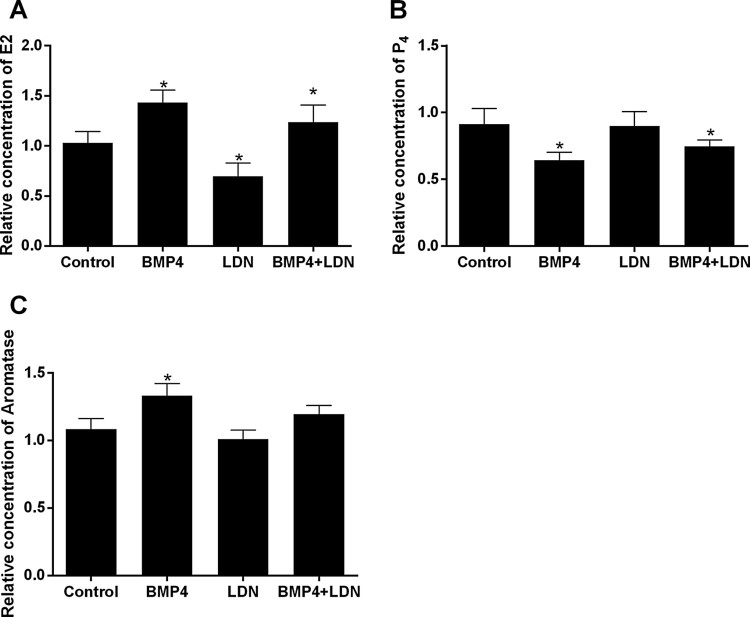
Effect of drug treatment on the secretion of E2, P4 and aromatase. After culture, hormone levels in the culture medium were measured by ELISA kit. Data are expressed as the mean ± S.D. (*N* = 3), *P < .05.

To investigate whether inhibition of BMP/Smad signaling pathway has an effect on hormone secretion, hormone (E_2_, P4 and aromatase) levels in the culture medium were measured by ELISA kit. Compare with control group, BMP-4 group decrease the secretion of P_4_, but increase the secretion of E_2_ and aromatase significantly (*P* < .05); LDN-193189 group decrease the secretion of E_2_ significantly, the difference was statistically significant (*P* < .05), it has no effect on P_4_ and aromatase; BMP-4 + LDN-193189 group increase the secretion of E_2_, decrease the secretion of P_4_, the difference was statistically significant (*P* < .05) but it has no significant influence on and aromatase (Figure 4(A–C)).

### LDN-193189 affects the expression of genes associated with ovulation by suppressing BMP/Smad signaling pathway

**Figure 5.**
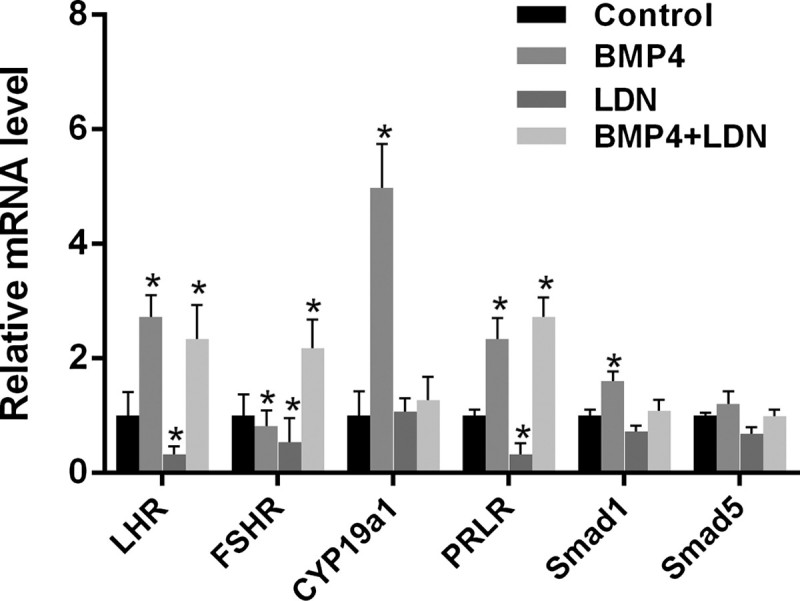
Expression of LHR, FSHR, CYP19a1, PRLR, Smad1 and Smad5 mRNA after drug treatment. Total RNA was extracted from mouse ovary granule cells and subjected to QRT-PCR to determine the mRNA levels. Data were normalized to Gapdh mRNA levels. Data are expressed as the mean ± S.D. (*N* = 3), *P < .05.

We choose genes (LHR, FSHR, CYP19a1, PRLR, Smad1 and Smad5) that associated with steroid hormones or participate in BMP/Smad signaling pathway to exam the mRNA levels. LHR, FSHR and PRLR gene expression in LDN-193189 group were significantly lower than that in the control group and BMP-4 group, and the difference was statistically significant (*P* < .05); Compare with control group, BMP-4 can promote the expression of LHR, CYP19a1, PRLR, and Smad1 significantly (*P* < .05), but LDN-193189 had no effect on the expression of them; There was no significant difference in the expression of Smad5 in the four groups; Interestingly, BMP-4 group and LDN-193189 group can inhibit the expression of FSHR, but BMP-4 + LDN-193189 increase the expression of FSHR (Figure 5).

### LDN-193189 affects the proliferation of ovary granule cells by suppressing BMP/Smad signaling pathway

**Figure 6.**
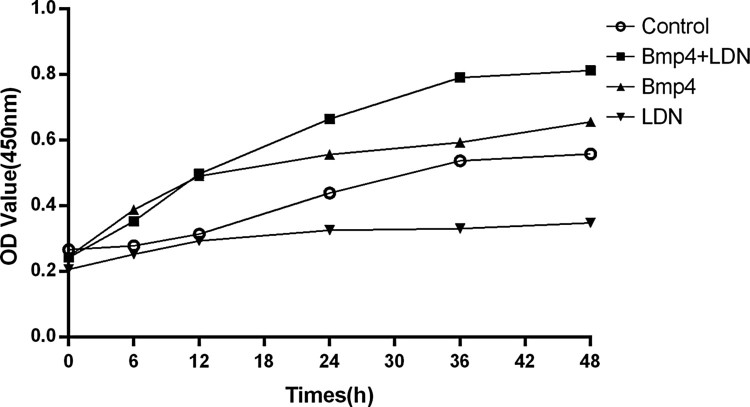
Growth curve of cultured ovarian granulosa cells. Cells were cultured 48 h either no additions (control), BMP-4 (100 μg/μl) or BMP-4 with LDN-193189 (100 nM). After culture, the absorbance at 450 nm was measured by a microplate reader. Data are expressed as the mean ± S.D. (*N* = 3).

Compare with control group, BMP-4 group could promote cell proliferation and LDN-193189 group inhibited cell proliferation, but interestingly, BMP-4 + LDN-193189group had more cells than BMP group (Figure 6).

## Discussion

Studies have shown that, first, extracellular signaling molecules BMP-4 is combined with BMPR-II　receptor, BMPR-I receptor activated by phosphorylated BMPR-II and forms a receptor complex with BMPR-II, then, activate I receptor protein kinase to make the intracellular Smads protein(such as smad1/5/9) phosphorylated, then the activated r-smads (mainly Smad1/5/9) combined with the common type of co-smad4 to form the Smad protein complex into the nucleus to regulate the transcription of the target gene (Drummond 2005). The substrates and downstream effectors of BMP type I receptors are critical for the activity of BMPs (Tsukamoto et al. 2014). BMP-4 is one of the bone morphogenetic proteins, it plays an important role in the process of embryonic development, and BMP-4 has important influence on female reproductive performance (Shimasaki et al. 1999; Nilsson & Skinner 2003).

Study confirms that, BMP-4, BMP-6 and BMP-7 particles can be used as cattle have follicular cavity cell proliferation of steroid and peptide secretion regulation autocrine/paracrine factor, they can effectively inhibit the secretion of male hormones induced by the follicular membrane cell and LH, and also play a role in the maintenance of progesterone secretion and cell number. Now, people have found that Smad9 is a new type of transcriptional regulator in bone morphogenetic protein signaling, activate the BMP/Smad signaling pathway could increase Smad9 expression, the over expression of Smad9 can inhibit the activity of BMP-4 by inhibiting the transcription of target genes (Tsukamoto et al. 2014), but the study on the function of Smad9 protein in ovarian GCs is rare, and the specific mechanism and signal pathway are unknown.

We used mouse ovary GCs as experimental materials to explore the effects of mouse ovarian GCs function and related gene expression by suppressing BMP/Smad signaling pathway. The mouse ovary GCs of the original culture were able to retain the cell characteristics to a maximum extent. There are many researches on vitro culture of ovarian GCs (Sasson et al. 2004; Park et al. 2010), we improved the experimental method according to the experimental condition, and found the method which is relatively suitable for us , it reduces the damage to the cell, keeps the cell morphology intact, and avoids mixing other cells, improving the survival and purity of GCs.

To explore the role of smad9 in signaling pathway, we control BMP/Smad signaling pathway by BMP-4 and LDN-193189(Cannon et al. 2010; Derwall et al. 2012; Lee et al. 2011; Mayeur et al. 2015; Yu et al. 2008). Our data showing that the phosphorylation of smad9 protein can be reduced by inhibiting the BMP/Smad signaling pathway (Figure 1), we examined the expression of mRNA and protein in smad9. The results show that BMP-4 promotes the expression of smad9 and LDN-193189 inhibits the expression of Smad9 significantly, BMP-4 + LDN-193189 can also promote the expression of smad9, the results are basically consistent with other people’s studies (Tsukamoto et al. 2014). Our findings indicate that smad9 may have important regulatory role in the growth of mouse ovary GCs growth and steroid hormone generation. We first studied the effects of BMP/Smad signaling pathway on the growth of mouse ovary GCs and steroid hormone secretion. Inhibit BMP/Smad signaling pathway and stimulate without exogenous FSH inhibited cell growth and decreased E_2_ secretion, but there was no significant effect on P_4_ and aromatase, results are not exactly the same as what we already know (Pierre et al. 2004; Yu et al. 2008). These differences may be caused by differences in species, different systems of culture, different dosage of drugs and so on. Furthermore, we know that, activating or inhibiting the signaling pathway reduces the expression of FSHR, but in BMP-4 + LDN-193189 group, the expression of FSHR is increased; our data in vitro results indicated that regulate BMP/Smad signaling pathway can directly affect cell proliferation, affects cell proliferation, but interestingly, the cell proliferation of the BMP-4 + LDN-193189 group was higher than that of BMP-4 group.

Taken together, our results show that the expression of smad9 gene in GCs can be inhibited by adding LDN-193189 to inhibit BMP/Smad signaling pathway, and Smad9 is positively correlated with the secretion of E_2_ and the transcription of LHR. According to the test results of hormone levels and gene transcription levels, we speculate that smad9 may participate in regulating E_2_ secretion and LHR transcription. At the same time, the expression of Smad9 may affect LHR transcription and cell proliferation, we speculate the possible mechanism is that Smad9 promote FSHR transcription and cell proliferation, but in turn, the over expression of Smad9 can inhibit FSHR expression and cell proliferation, it indicates that smad9 may have negative feedback regulation this result is consistent with the research of Tsukamoto et al. (2014). But these specific regulatory mechanisms are not clear and that is what we are going to do next.
